# Association of personality with the development and persistence of obesity: a meta-analysis based on individual–participant data

**DOI:** 10.1111/obr.12007

**Published:** 2012-11-26

**Authors:** M Jokela, M Hintsanen, C Hakulinen, G D Batty, H Nabi, A Singh-Manoux, M Kivimäki

**Affiliations:** 1 Institute of Behavioral Sciences, University of HelsinkiHelsinki, Finland; 2 Research Department of Epidemiology and Public Health, University College LondonLondon, UK; 3 Helsinki Collegium for Advanced Studies, University of HelsinkiHelsinki, Finland; 4 Centre for Cognitive Ageing and Cognitive Epidemiology, Department of Psychology, University of EdinburghEdinburgh, UK; 5 INSERM U1018, AP-HPParis, France; 6 Finnish Institute of Occupational HealthHelsinki, Finland

**Keywords:** Longitudinal analysis, meta-analysis, obesity, personality

## Abstract

Personality is thought to affect obesity risk but before such information can be incorporated into prevention and intervention plans, robust and converging evidence concerning the most relevant personality traits is needed. We performed a meta-analysis based on individual–participant data from nine cohort studies to examine whether broad-level personality traits predict the development and persistence of obesity (*n* = 78,931 men and women; mean age 50 years). Personality was assessed using inventories of the Five-Factor Model (extraversion, neuroticism, agreeableness, conscientiousness and openness to experience). High conscientiousness – reflecting high self-control, orderliness and adherence to social norms – was associated with lower obesity risk across studies (pooled odds ratio [OR] = 0.84; 95% confidence interval [CI] = 0.80–0.88 per 1 standard deviation increment in conscientiousness). Over a mean follow-up of 5.4 years, conscientiousness predicted lower obesity risk in initially non-obese individuals (OR = 0.88, 95% CI = 0.85–0.92; *n* = 33,981) and was associated with greater likelihood of reversion to non-obese among initially obese individuals (OR = 1.08, 95% CI = 1.01–1.14; *n* = 9,657). Other personality traits were not associated with obesity in the pooled analysis, and there was substantial heterogeneity in the associations between studies. The findings indicate that conscientiousness may be the only broad-level personality trait of the Five-Factor Model that is consistently associated with obesity across populations.

## Introduction

The obesity epidemic has reached alarming proportions in Western countries. In the United States, 34% of the population is obese 1, and in many European countries, including Spain, Italy and the United Kingdom, the prevalence of obesity is over 20% 2. With the exception of bariatric surgery for morbidly obese, obesity treatments have poor long-term maintenance 3,4. Intervention programs usually result in an average weight loss of 3–5 kg, which many regain within a few years after the treatment 5. To date, relatively little is known about factors that explain individual differences in long-term obesity risk or the likelihood of reversion from obese to non-obese 4.

Psychological dispositions, indicated by measures of personality, have been implicated in obesity risk 6. While early studies on personality differences between obese and non-obese individuals found no consistent differences 7,8, these had considerable methodological limitations, including small sample sizes and non-standardized personality measures. Recent studies with stronger study designs have found obesity to be associated with various personality traits 9–15. Many of these findings have not been replicated across studies 13,14 and different studies have reported conflicting findings for the same personality traits, including extraversion 10,11,15 and agreeableness 13,14. Hence, it remains unclear which personality dimensions are involved in the development of obesity and which dimensions are associated with obesity only secondarily or by chance. Before information on personality dispositions can be successfully incorporated into prevention and intervention plans, robust and converging evidence concerning whether personality traits are related to obesity is needed.

In the present study, we examined cross-sectional and longitudinal associations between personality and obesity in nine cohorts from the United States, the United Kingdom, Germany and Australia. Broad-level personality traits were assessed using different inventories of the Five-Factor Model of personality (FFM, also known as the Big Five), which has emerged as the most robust and widely accepted model to describe main dimensions of human personality 16. By pooling data across nine cohorts with a total of almost 80,000 participants, we were able to evaluate personality–obesity associations with a greater precision than has been previously possible.

## Methods and materials

### Study participants

We searched the data collections of the Inter-University Consortium for Political and Social Research (http://www.icpsr.umich.edu/icpsrweb/ICPSR/) and the Economic and Social Data Service (http://www.esds.ac.uk) to identify eligible large-scale cohort studies on personality and obesity. The studies had to include information on participant's height and weight, and on personality assessed with at least the brief 15-item questionnaire based on the Five-Factor Model. Study participants were drawn from the Add Health (ADDHEALTH) Study; the British Household Panel Survey (BHPS); the German Socio-Economic Panel Study; the Household, Income and Labour Dynamics in Australia; the Health and Retirement Study (HRS); the Midlife in the United States Study; the National Child Development Study (NCDS); and the Wisconsin Longitudinal Study Graduate Sample (WLSG) and Wisconsin Longitudinal Study Sibling Sample. These are all well-characterized cohort studies. Details of the studies are reported in online supplementary material. Although they are all longitudinal studies, ADDHEALTH, BHPS and NCDS did not have follow-up data on obesity after the assessment of personality, so these cohorts were included only in the cross-sectional analyses.

### Measurements

In all studies, the FFM personality traits were assessed using standardized questionnaire instruments 17–19. The model postulates five broad-level personality traits that sum up individual variation in several more specific personality dispositions: *extraversion* (e.g. social assertiveness, sociability, sensitivity to positive emotions), *neuroticism* (e.g. low emotional stability, sensitivity to negative emotions, anxiety proneness), *agreeableness* (e.g. cooperativeness, altruism, trust towards other people), *conscientiousness* (e.g. self-control, orderliness, adherence to social norms) and *openness to experience* (e.g. curiosity, broad-ranging interests, open-mindedness). Height and weight were objectively measured in ADDHEALTH and self-reported in other studies. Data on other study covariates were derived from self-reported questionnaires and interviews.

### Statistical analysis

We examined cross-sectional associations between the five personality traits and obesity in the total sample and within different subgroups using logistic regression (0 = non-obese, 1 = obese). Odds ratios (ORs) were presented calculated for personality z-scores (standard deviation [SD] = 1). We also examined whether the cross-sectional associations replicated in longitudinal analysis, after controlling for baseline obesity, and whether the statistically significant associations followed a graded dose–response pattern across personality score quintiles. To determine whether baseline personality was associated with development and persistence of obesity, we examined the longitudinal associations stratified by obesity status at baseline. As a test of reverse causality, we examined whether obesity status at baseline predicted change in personality at follow-up. In all the analyses, we first examined the association separately within each cohort, and then conducted a meta-analysis assuming random effect across studies. We calculated the ORs per 1 SD increment in each personality trait and adjusted the estimates for sex, age at baseline, and ethnicity/nationality (0 = majority, i.e. in most cohort non-Hispanic Caucasians; 1 = other). Longitudinal analyses were further adjusted for follow-up period in months. For the analysis of personality change, personality scores were standardized against baseline personality score in each study so that the change scores could be combined in meta-analysis. Subgroup differences were tested by first fitting the models separately by subgroups in the individual studies, pooling these results together by subgroup with meta-analysis, and examining heterogeneity across the effects sizes estimated for the subgroups. All analyses were conducted using STATA version 12.1, StataCorp, College Station, TX, USA (metan command for meta-analysis).

## Results

The total sample consisted of 78,931 mostly middle-aged participants (age range 15–104, mean age 50.4 years; 53.2% women). Obesity prevalence in the included studies varied between 18 and 37% (Table 1). Mean follow-up period across the studies (*n* = 43,638) with a longitudinal element was 5.4 years.

**Table 1:** Characteristics of included studies

	ADDHEALTH	BHPS	GSOEP	HILDA	HRS	MIDUS	NCDS	WLSG	WLSS
Number of participants									
Cross-sectional analysis	4,957	7,738	18,387	9,434	13,716	5,997	8,330	6,515	3,857
Longitudinal analysis	–	–	12,258	7,646	12,452	3,559	–	5,082	2,641
Age	29.0 (1.8)	46.6 (17.5)	47.4 (17.3)	44.7 (17.7)	67.3 (10.4)	46.9 (12.9)	50.3 (0.5)	54.1 (0.5)	53.1 (7.3)
Sex									
Men	45.9 (2,275)	50.7 (3,921)	47.9 (8,807)	47.1 (4,440)	41.2 (5,648)	48.0 (2,876)	48.9 (4,073)	46.9 (3,053)	47.3 (1,825)
Women	54.1 (2,682)	49.3 (3,817)	52.1 (9,580)	52.9 (4,994)	58.8 (8,068)	52.0 (3,121)	51.1 (4,257)	53.1 (3,462)	52.7 (2,032)
Education									
Primary	33.6 (1,664)	28.6 (2,212)	16.2 (2,817)	34.0 (3,212)	18.5 (2,537)	8.7 (518)	18.2 (1,519)	–	5.1 (197)
Secondary	53.5 (2,651)	56.6 (4,383)	61.2 (10,670)	44.1 (4,158)	55.2 (7,563)	59.1 (3,536)	61.8 (5,146)	72.0 (4,692)	64.0 (2,470)
Tertiary	13.0 (642)	14.8 (1,142)	22.6 (3,938)	21.9 (2,064)	26.3 (3,601)	32.3 (1,930)	20.0 (1,665)	28.0 (1,823)	30.9 (1,190)
Follow-up time (months)	–	–	48.1 (1.6)	48.0 (1.3)	35.4 (16.0)	107.5 (6.3)	–	134.0 (4.2)	135.8 (6.6)
Marital status									
Married/cohabiting	41.5 (2,056)	55.3 (4,277)	60.2 (11,063)	61.2 (5,775)	64.0 (8,777)	67.9 (4,069)	70.3 (5,852)	83.2 (5,419)	80.9 (3,093)
Single	58.5 (2,901)	44.7 (3,460)	39.8 (7,324)	38.8 (3,659)	36.0 (4,937)	32.1 (1,925)	29.7 (2,478)	16.8 (1,096)	19.1 (732)
Baseline obesity									
Non-obese	63.1 (3,129)	76.2 (5,893)	84.0 (15,437)	79.2 (7,469)	69.1 (9,480)	80.9 (4,850)	75.9 (6,319)	81.8 (5,327)	82.1 (3,167)
Obese	36.9 (1,828)	23.8 (1,845)	16.0 (2,950)	20.8 (1,965)	30.9 (4,236)	19.1 (1,147)	24.1 (2,011)	18.2 (1,188)	17.9 (690)
Follow-up obesity									
Non-obese	–	–	81.4 (10,440)	76.7 (5,865)	69.9 (8,710)	71.9 (2,564)	–	75.0 (3,811)	75.9 (2,004)
Obese	–	–	18.6 (2,383)	23.3 (1,781)	30.1 (3,756)	28.1 (1,002)	–	25.0 (1,271)	24.1 (637)
Ethnicity/nationality									
Majority	71.9 (3,564)	87.9 (6,799)	93.0 (17,107)	86.5 (8,156)	78.0 (10,696)	89.3 (5,358)	98.0 (8,162)	100.0 (6,515)	100.0 (3,857)
Minority	28.1 (1,393)	12.1 (939)	7.0 (1,280)	13.5 (1,278)	22.0 (3,020)	10.7 (639)	2.0 (168)	–	–

Note: Because of missing data in covariates, numbers of covariate frequencies may not add up to the total number of participants with personality data.

ADDHEALTH, National Longitudinal Study of Adolescent Health; BHPS, British Household Panel Survey; GSOEP, German Socio-Economic Panel Study; HILDA, Household, Income and Labour Dynamics in Australia; HRS, Health and Retirement Study; MIDUS, Midlife in the United States; NCDS, National Child Development Study; WLSG, Wisconsin Longitudinal Study Graduate Sample; WLSS, Wisconsin Longitudinal Study Sibling Sample.

### Cross-sectional analysis

**Figure 1:**
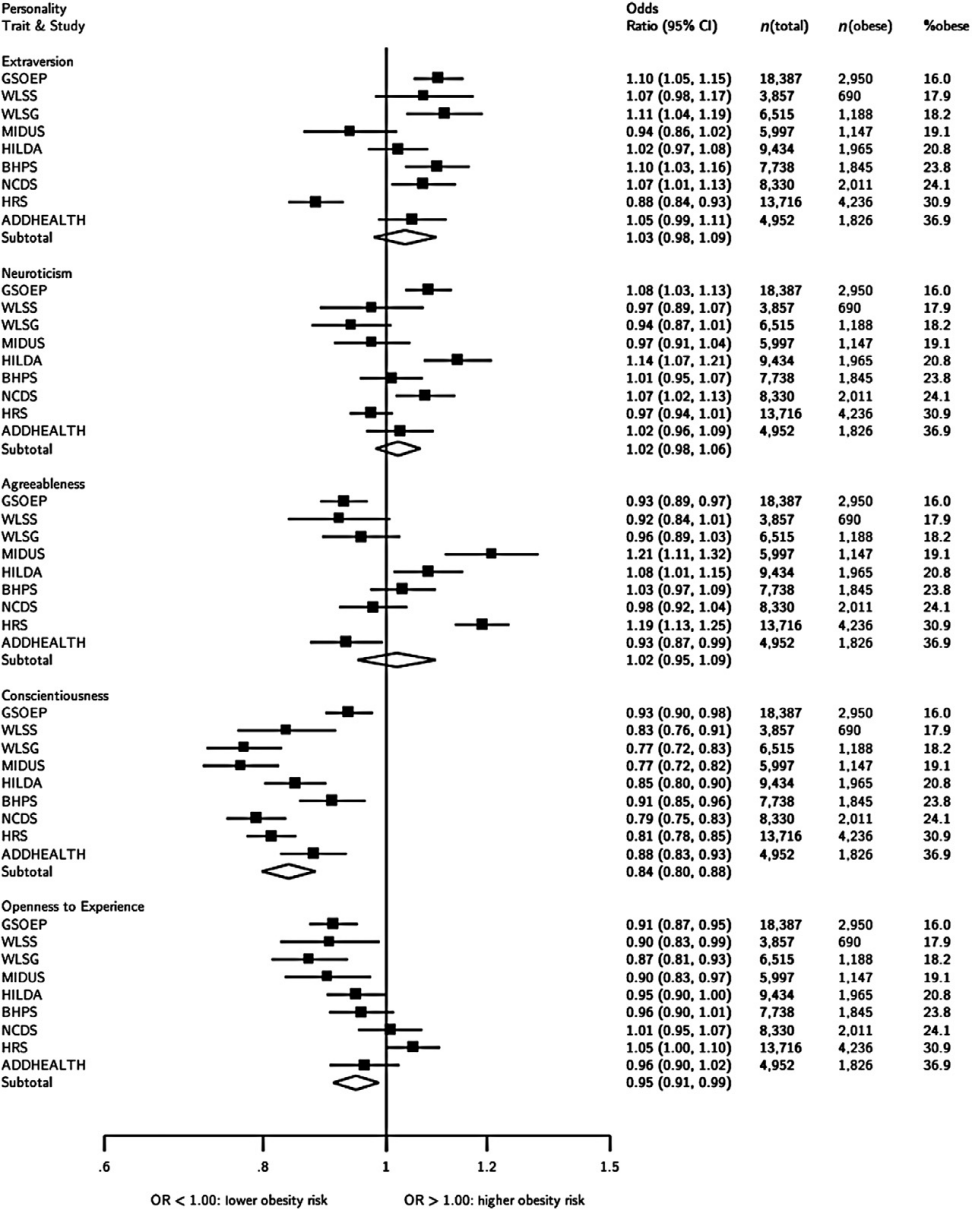
Cross-sectional associations between Five-Factor Model personality traits and obesity at baseline. Values are odds ratios per 1 standard deviation increment in personality trait. Personality traits are adjusted for each other in addition to sex, age and race/ethnicity. Studies are listed in decreasing order of study-specific obesity prevalence. ADDHEALTH, National Longitudinal Study of Adolescent Health; BHPS, British Household Panel Survey; GSOEP, German Socio-Economic Panel Study; HILDA, Household, Income and Labour Dynamics in Australia; HRS, Health and Retirement Study; MIDUS, Midlife in the United States; NCDS, National Child Development Study; WLSG, Wisconsin Longitudinal Study Graduate Sample; WLSS, Wisconsin Longitudinal Study Sibling Sample.

Figure 1 shows that greater conscientiousness was consistently associated with lower obesity risk across studies in the cross-sectional analysis, with pooled OR = 0.84 per SD increment in conscientiousness (95% confidence interval [CI] = 0.80–0.88). Thus, compared to individuals with high conscientiousness (1 SD above the mean), individuals with low conscientiousness (1 SD below the mean) had 38% higher odds [ = (1/0.84 − 1) × 2 × 100] of obesity adjusted for other personality traits, sex, age and race/ethnicity. Higher openness to experience was associated with lower odds of obesity (pooled OR = 0.95; CI = 0.91–0.99) but this association disappeared when adjusted for education (OR = 0.99; CI = 0.95–1.04). The longitudinal models described below were therefore adjusted for education.

While individual studies suggested some statistically significant cross-sectional associations for the other four personality traits, none of the pooled ORs suggest an overall association. There was considerable heterogeneity in the associations across studies as indicated by I^2^ values of 87% (95% CI 78–93%) for extraversion, 78% (58–88%) for neuroticism, 92% (86–95%) for agreeableness, 84% (72–91%) for conscientiousness and 77% (56–88%) for openness to experience.

To examine whether the association between conscientiousness and obesity was modified by any of the socio-demographic variables included in the study, we examined the associations by various subgroups (Supporting Information Figure S1). The association was stronger in women (OR = 0.81; CI = 0.77–0.85) than in men (OR = 0.88; CI = 0.84–0.92) but did not vary by age, ethnicity, region, marital status or education. We also examined the subgroup differences with other personality traits (Supporting Information Table S1). There were three statistically significant subgroup differences (*P* < 0.05): higher extraversion was associated with higher obesity risk in men (OR = 1.09, 1.04–1.14) but not in women (OR = 0.99, 0.92–1.06); higher extraversion was associated with higher obesity risk in European samples (OR = 1.09, 1.06–1.12) but not in American (OR = 1.00, 0.91–1.11) or Australian (OR = 1.02, 0.97–1.08) samples; and higher neuroticism was associated with higher obesity risk in Australian (OR = 1.14, 1.07–1.21) and European (OR = 1.06, 1.02–1.10) but not in American (OR = 0.98, 0.95–1.00) samples. However, only the interaction effect between neuroticism and geographic area remained significant after taking into account multiple testing (Bonferroni-corrected *P* value < 0.001, adjusted for 30 interaction tests).

### Longitudinal analysis

Adjusted for baseline obesity status, the longitudinal associations between baseline personality and subsequent obesity risk were very similar as in the cross-sectional analysis. Table 2 shows the associations for conscientiousness. This association was similar in individuals who were non-obese or obese at baseline, indicating that high conscientiousness was associated with lower risk of developing and lower persistence of obesity over time. In the obese, higher conscientiousness was associated with a higher likelihood of reversion to non-obese at follow-up (OR per 1 SD increase in conscientiousness = 1.08, 95% CI = 1.01–1.14). Details of the longitudinal analyses for other personality traits are reported in Supporting Information Figures 2. Similar to the cross-sectional association, the longitudinal association was stronger in women (pooled OR = 0.86, 0.81–0.90) than in men (pooled OR = 0.95, 0.90–1.00, *P* = 0.05).

**Figure 2:**
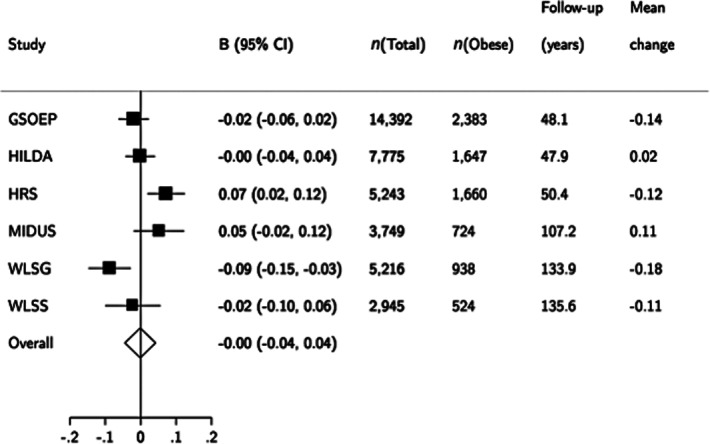
Baseline obesity predicting personality change in conscientiousness between baseline and follow-up wave (combined *n* = 39,320). Values are standardized regression coefficients for conscientiousness change (standardized with the baseline conscientiousness level within each sample, standard deviation = 1). Mean change gives the average change in conscientiousness in the sample. GSOEP, German Socio-Economic Panel Study; HILDA, Household, Income and Labour Dynamics in Australia; HRS, Health and Retirement Study; MIDUS, Midlife in the United States; WLSG, Wisconsin Longitudinal Study Graduate Sample; WLSS, Wisconsin Longitudinal Study Sibling Sample.

**Table 2:** Longitudinal associations between baseline conscientiousness and subsequent obesity risk

	All	Non-obese at baseline	Obese at baseline
Individual cohorts			
GSOEP	0.93 (0.87, 1.00)	0.91 (0.83, 0.99)	0.97 (0.86, 1.10)
WLSS	0.87 (0.77, 0.99)	0.85 (0.74, 0.98)	0.99 (0.76, 1.30)
WLSG	0.88 (0.80, 0.96)	0.86 (0.78, 0.95)	0.94 (0.78, 1.14)
MIDUS	0.93 (0.83, 1.03)	0.91 (0.81, 1.02)	0.99 (0.78, 1.26)
HILDA	0.91 (0.84, 0.99)	0.91 (0.82, 1.02)	0.91 (0.79, 1.05)
HRS	0.88 (0.81, 0.94)	0.86 (0.77, 0.96)	0.89 (0.80, 0.99)
Pooled	0.90 (0.87, 0.93)	0.89 (0.85, 0.92)	0.93 (0.88, 0.99)
*n* (total)	43,638	33,981	9,657

Note: Values are standardized odds ratios (and 95% confidence intervals) adjusted for baseline obesity status, the other four personality traits, educational level, sex, age, follow-up length, ethnicity/nationality. The pooled estimate was calculated using random-effect meta-analysis. See Supporting Information Figures S2–S4 for details.

ADDHEALTH, National Longitudinal Study of Adolescent Health; BHPS, British Household Panel Survey; GSOEP, German Socio-Economic Panel Study; HILDA, Household, Income and Labour Dynamics in Australia; HRS, Health and Retirement Study; MIDUS, Midlife in the United States; NCDS, National Child Development Study; WLSG, Wisconsin Longitudinal Study Graduate Sample; WLSS, Wisconsin Longitudinal Study Sibling Sample.

The longitudinal association between conscientiousness and obesity in the total sample followed a graded dose–response pattern across quintiles of conscientiousness, so that the odds of obesity decreased in order of increasing quintiles were 1.00 (lowest quintile in the reference group), 0.94 (0.72–1.24), 0.87 (0.67–1.12), 0.79 (0.60–1.03) and 0.77 (0.60–0.98). Thus, individuals in the lowest conscientiousness quintile had 30% higher odds (=1/0.77) of being obese compared to individuals in the highest quintile. Details for the quintile regressions for all personality traits are reported in Supporting Information Figure S5.

### Personality change (test for reverse causality)

In the pooled data, baseline obesity status did not predict change in conscientiousness over time (Fig. 2), although there was some heterogeneity between studies as the significant associations observed in HRS and WLSG studies were in the opposite directions.

## Discussion

The purpose of the present individual–participant meta-analysis was to examine the links between personality dispositions and obesity more comprehensively than has hitherto been the case. Data from 9 cohorts with almost 80,000 participants show that the personality trait conscientiousness is robustly associated with the development and persistence of obesity. Individuals with high conscientiousness are described as self-disciplined, task oriented and well organized, whereas low conscientiousness is characterized by poor self-control, impulsivity and lack of long-term planning 16. Compared to individuals with low conscientiousness (1 SD below the mean), individuals with high conscientiousness (1 SD above the mean) have almost 40% lower odds of being obese. This association was observed in cross-sectional and longitudinal analysis with an average follow-up time of 7 years, and it is somewhat stronger in women than in men, and in Caucasians than in non-Caucasians. We found no evidence to suggest that this association might have been attributable to reverse causation bias. Although some associations were observed for the other four personality traits in individual studies, only conscientiousness was consistently associated with obesity.

A drawback of the study setting was that the participants included mostly adults with a mean age of 53 years. At this life stage, obesity status has already stabilized substantially, which prevented us from examining early developmental patterns between personality and obesity. It would be informative to examine the role of personality traits in obesity development from childhood and adolescence onwards 20. A further limitation is that the personality measures used in the studies were relatively brief and only measured the five broad-level personality traits but not any subscales of these traits. It is possible that the broad-level personality traits are too general to capture some of the more specific associations between narrow-level personality traits and obesity risk. Moreover, some of the observed heterogeneity in effect sizes across studies may be explained by differences in personality instruments between studies, although these differences are unlikely to explain directly contrasting results (positive association in one study, negative association in another) because all the subscales of the personality traits are naturally positively correlated with each other. Furthermore, despite the differences in personality instruments, the association between conscientiousness and obesity was consistent across studies.

A recent literature-based review concluded that personality traits may be unrelated to obesity risk and weight management 3. However, many of the studies testing these associations have focused on specific emotional or cognitive styles rather than personality traits related to conscientiousness, and therefore may not have addressed the correct personality dimensions. The relevance of conscientiousness for health has been recognized recently with studies on mortality 21 and health behaviours 22. The association between conscientiousness and obesity observed here is plausible because conscientiousness has been associated with a broad range of health behaviours, including physical activity, adherence to medication, non-smoking, less risky behaviours and healthy dietary patterns, among other behaviours 22,23.

Results from observational cohort studies may not be directly generalizable to intervention studies, but there is evidence to suggest that conscientiousness is related to success of specific treatment approaches 24,25. For example, one study found that individuals with high conscientiousness lost more weight with pharmacological obesity treatment using orlistat 24, a drug taken three times daily with each meal to reduce the absorption of dietary fat. Our findings lend support for conscientiousness being a prognostic factor for the reversion of obesity to non-obesity in initially obese individuals; obesity was less persistent in individuals with high conscientiousness. This supports the notion that assessment of consciousness may help identify the most suitable and effective weight management therapies for individuals with different personality dispositions.

Several previous studies have demonstrated a bidirectional association between obesity and self-reported or clinical measures of depression; obesity appears to increase the risk of depression and depression appears to increase the risk of obesity 26. Although neuroticism is strongly associated with depression risk 27, we found no evidence to suggest that neuroticism would be associated with obesity risk. This suggests that the obesity–depression association observed in previous studies is unlikely to reflect underlying personality differences between obese and non-obese individuals. However, subgroup analyses suggested that obesity may be associated with higher neuroticism in European countries and in Australia but not in the United States. Further research is required to identify potential contextual or cultural factors that might explain these differences.

In conclusion, the current data show that conscientiousness is robustly associated with obesity risk in general populations from the United States, the United Kingdom, Germany and Australia. Assessment of conscientiousness might help identify obese individuals for whom weight loss is particularly unlikely without extra support or for whom some treatment strategies work better than others. Other broad-level personality traits do not appear to be important for obesity risk, at least in adulthood, suggesting that if information on broad-level personality traits was incorporated into prevention and intervention plans, measurement of conscientiousness would be most relevant. Future studies should concentrate on measures of conscientiousness and different lower level personality facets in evaluating the value of personality in individualized obesity prevention and treatment strategies.

## Supporting Information

**Figure S1.** Cross-sectional association between conscientiousness and obesity by subgroups (standardized odds ratios and 95% confidence intervals). The p-values for heterogeneity (I^2^) indicate statistical significance of group differences within subgroups.

**Figure S2.** Longitudinal associations between baseline personality and subsequent obesity in the total sample (*n* = 43,638) adjusted for baseline obesity status, the other four personality traits, educational level, sex, age, follow-up length, ethnicity/nationality.

**Figure S3.** Longitudinal associations between baseline personality and subsequent obesity in non-obese individuals at baseline (*n* = 33,981) adjusted for the other four personality traits, educational level, sex, age, follow-up length, ethnicity/nationality.

**Figure S4.** Longitudinal associations between baseline personality and subsequent obesity in obese individuals at baseline (*n* = 9,657) adjusted for the other four personality traits, educational level, sex, age, follow-up length, ethnicity/nationality.

**Figure S5.** Pooled longitudinal associations between baseline personality traits and subsequent obesity when personality scores are categorized into quintiles in each study (I = lowest quintile, the reference category, V = highest quintile). All associations are adjusted for obesity status at baseline, sex, age, educational level, follow-up length and ethnicity/nationality. *n* = 43,638.

**Table S1.** Subgroup analyses of cross-sectional associations between personality traits and obesity.
