# Supplementary material for: Association of personality with the development and persistence of obesity: a meta-analysis based on individual–participant data
Source: Obes Rev. 2012 Nov 26;14(4):315–23. doi: 10.1111/obr.12007 (PMC3717171; doi:10.1111/obr.12007)
Supplement: Supplementary file 1 [file obr0014-0315-SD1.docx]

**Association of Personality with the Development and Persistence of Obesity: A Meta-Analysis Based on Individual-Participant Data**

Markus Jokela, Mirka Hintsanen, Christian Hakulinen, G. David Batty, Hermann Nabi, Archana Singh-Manoux, Mika Kivimäki

Supplementary material

1. Description of study cohorts
2. Supplementary Figures S1–S5
3. Supplementary Table S1

National Longitudinal Study of Adolescent Health (ADDHEALTH)

The National Longitudinal Study of Adolescent Health (Add Health) is a longitudinal study of a nationally representative sample of adolescents in grades 7-12 in the United States during the 1994-1995 school year.^1^ Add Health combines longitudinal survey data on respondents' social, economic, psychological and physical well-being with contextual data on the family, neighborhood, community, school, friendships, peer groups, and romantic relationships, providing unique opportunities to study how social environments and behaviors in adolescence are linked to health and achievement outcomes in young adulthood. Wave I of the study began collecting data during the 1994–1995 school year, when the participants were in grades 7–12, and the most recent wave of data (Wave IV) was collected in 2008 when the participants were between the ages of 24–32 years old.

**Personality** was measured in the 4^th^ data collection wave in 2008 using a 20-item Five Factor Model personality instrument, 4 items per trait rated on a 5-point rating scale. Cronbach alpha reliabilities were 0.71 for extraversion, 0.63 for neuroticism, 0.70 for agreeableness, 0.65 for conscientiousness, and 0.65 for openness to experience. Personality scales were calculated for individuals with no missing values in the 4 items, resulting in a sample of 5,026 participants with full personality data at baseline.

**Height** and **weight** were measured in a medical examination, and **body mass index (BMI)** was calculated from these data with the standard formula of BMI=weight in kilograms divided by squared height in meters. **Obesity** was determined as BMI≥30. Information on **marital status** (0=single, 1=married/cohabiting) was derived from the participants’ self-reports. Data on **race/ethnicity** was based on participants’ self-reports and was coded as a dichotomous variable (0=white, non-Hispanic; 1=other). **Educational level** was determined on the basis of the highest achieved grade (0=primary education, 1=secondary education, 3=tertiary education).

Study website:

<http://www.cpc.unc.edu/projects/addhealth>

British Household Panel Survey (BHPS)

The British Household Panel Survey (BHPS) is a longitudinal survey of a nationally representative sample of over 5000 British households with annual follow-ups. ^2^ The original cohort included 10,264 individuals aged 16-97 at baseline in 1991, and was based on a clustered, stratified sample of addresses throughout Great Britain south of the Caledonian Canal (excluding North of Scotland and Northern Ireland). New participants have been included in the sample over the years if they are born to original sample member, if they have moved into a household in the original sample, or if a member of the original sample moves into a new household with one or more new people. In addition, the sample was enriched with additional recruitment of participants at waves 9 and 11, from Scotland and Wales, and from Northern Ireland, respectively, so extending the sample to cover the whole UK. The most recent (18^th^) follow-up of the BHPS was carried out in 2008-2009, after which the cohort has become part of the larger Understanding Society Study.

**Personality** was assessed in the 15^th^ data collection wave in 2005 using a brief, 15-item version of the Big Five Inventory (BFI)^3,4^ with three items assessing each personality trait, rated on a 7-point scale. Cronbach alpha reliabilities were 0.54 for extraversion, 0.68 for neuroticism, 0.53 for agreeableness, 0.51 for conscientiousness, and 0.67 for openness to experience. Personality scales were calculated for individuals with no missing items in the scale, resulting in 13,825 participants with full personality data at baseline.

**Height** and **weight** were self-reported by the participants in the 16^th^ data collection wave in 2006. Although this was 1 year after the measurement of personality, these data were treated as cross-sectional because no additional data on height and weight at later data collection waves were available for the purpose of longitudinal analysis. **Body mass index (BMI)** was calculated from these data with the standard formula of BMI=weight in kilograms divided by squared height in meters. **Obesity** was determined as BMI≥30. Information on **marital status** (0=single, 1=married/cohabiting) was derived from the participants’ self-reports. Data on **race/ethnicity** was based on participants’ self-reports and was coded as a dichotomous variable (0=white, non-Hispanic; 1=other). **Educational level** was determined on the basis of the highest achieved grade (0=primary education, 1=secondary education, 3=tertiary education).

Study website:

<http://www.esds.ac.uk/longitudinal/access/bhps/L33196.asp>

<http://www.understandingsociety.org.uk/>

German Socio-Economic Panel Study (GSOEP)

The German Socio-Economic Panel Study (GSOEP, v27) is a longitudinal study of private households ^5,6^ The study started in 1984 in West Germany with two subsamples: Sample A, the main sample, covering the population of private households, and Subsample B, which oversampled the “guest worker households” with Turkish, Spanish, Italian, Greek and Yugoslavian household heads. The original sample included 5921 households and 12,245 individual respondents. Several additional samples have subsequently been integrated in the study, including a sample of Germans from the late East Germany in 1990 (2,179 households; 4,453 individuals), an immigrant sample in 1994/1995 (522 households; 1,078 individuals), a refreshment sample of existing subsamples in 1998 (1,056 households; 1,910 individuals), an “innovation” subsample again covering all existing subsamples in 2000 (6,043 households; 10,880 individuals), a high-income subsample of households with net earnings more than 4500 euros/month in 2002 (1,224 households; 2,671 individuals), a second refreshment sample covering all existing subsamples in 2006 (1,506 households; 2,616 individuals), and an “incentive” sample covering all existing subsamples in 2009 (1,531 households; 2,509 individuals). All household members aged 17 years or older are invited for interview, which are carried out annually. Altogether, a total of 34,881 individuals have participated in the study at least in one study wave.

**Personality** was assessed in the 22^nd^ data collection wave in 2005 using the 15-item version of the Big Five Inventory (BFI)^3,4,7^ with three items assessing each personality trait, rated on a 7-point scale. Cronbach alpha reliabilities were 0.66 for extraversion, 0.60 for emotional stability, 0.51 for agreeableness, 0.62 for conscientiousness, and 0.63 for openness to experience. Personality scales were calculated for individuals with no missing items in the scale, resulting in 20,434 participants with full personality data at baseline.

Data on **race/ethnicity** was based on participants’ self-reports and was coded as a dichotomous variable (0=white, non-Hispanic; 1=other). **Educational level** was determined on the basis of the highest achieved grade (0=primary education, 1=secondary education, 3=tertiary education). **Height** and **weight** were self-reported by the participants in the 23^rd^ data collection wave in 2006. Although this was 1 year after the measurement of personality, these data were treated as cross-sectional because no additional data on height and weight at later data collection waves were available for the purpose of longitudinal analysis. **Body mass index (BMI)** was calculated from these data with the standard formula of BMI=weight in kilograms divided by squared height in meters. **Obesity** was determined as BMI≥30.

Study website:

http://www.diw.de/en/soep

Household, Income and Labour Dynamics in Australia (HILDA) Survey

The Household, Income and Labour Dynamics in Australia (HILDA) Survey is a household-based panel study which began in 2001, developed particularly to collects information about economic and subjective well-being, labour market dynamics and family dynamics.^8^ The survey began with a large national probability sample of Australian households occupying private dwellings (n=7,682 households with 19,914 individuals at baseline). All members of the households providing at least one interview in wave 1 form the basis of the panel to be pursued in each subsequent wave. Interviews are conducted annually with all adult members of each household. The sample has been gradually extended to include any new household members resulting from changes in the composition of the original households. From wave 9, new household members that arrived in Australia for the first time after 2001 were also added to the sample. Up to wave 10 carried out in 2010, a total of 28,547 individuals had participated in the study at least in one study wave.

**Personality** was assessed in wave 5 in 2005 using a 36-item Five Factor Personality self-reported inventory based on the Goldberg’s Big Five Markers Scale,^9^ with 8 items for extraversion (α=0.77), 7 items for neuroticism (α=0.79), 7 items for agreeableness (α=0.77), 7 items for conscientiousness (α=0.79), and 6 items for openness to experience (α=0.73; the original item “traditional” was omitted from the scale because of a very low factor loading of 0.03 and a very low correlation of 0.02 between the item and a scale constructed from the rest of the items). The participants rated the items on a 7-point scale (1=Does not describe me at all, 7=Describes me very well). Personality sum scales were calculated for individuals with no more than 1 missing item in the scale, resulting in 11,091 individuals with full personality data at wave 5.

**Height** and **weight** were self-reported by the participants at wave 6 (i.e., one year after personality assessment), and **body mass index (BMI)** was calculated from these data with the standard formula of BMI=weight in kilograms divided by squared height in meters. **Obesity** was determined as BMI≥30. Data on **race/ethnicity** was based on participants’ self-reports and was coded as a dichotomous variable (0=white, non-Hispanic; 1=other). **Educational level** was determined on the basis of the highest achieved grade (0=primary education, 1=secondary education, 3=tertiary education).

Study website:

<http://www.melbourneinstitute.com/hilda/>

Health and Retirement Study (HRS)

The HRS is a nationally representative longitudinal study of more than 30,000 individuals representing the U.S. population older than 50 years.^10^ Telephone or in-person interviews are conducted every 2 years, administered under the National Institute of Aging (NIA) and the University of Michigan’s Institute for Social Research. As of 1998, the HRS consists of 4 sources of data collection: (1) The original HRS began as two distinct surveys that were merged in 1998, and are. The original HRS was initially administered in 1992 to a nationally representative sample of Americans born in the years 1931 through 1941. In the case of married couples, both spouses (including spouses who were younger than 51 or older than 61) were also interviewed; (2) The second survey, originally referred to as the Study of Assets and Health Dynamics Among the Oldest Old (AHEAD), was first administered in 1993 to a nationally representative sample of Americans born in 1923 or earlier (n=8,000) and merged with the HRS in 1998. In the case of married couples, interviews were conducted with both spouses; (3) In 1998, a subsample of individuals born between 1924 and 1930, referred to as Children of the Depression Age (CODA) was added to HRS; (4) Another subsample consisting of people born between 1942 and 1947 (War Baby cohort) was added to replenish the sample of people in their early 50s as the original HRS cohort aged. The Health Sciences Institutional Review Board at the University of Michigan approved the HRS.

**Personality** was measured using a self-reported instrument adapted from the MIDUS study^11^ with 5 items for extroversion (α=0.74), 4 items for neuroticism (α=0.63), 5 items for agreeableness (α=0.78), 5 items for conscientiousness (α=0.63), and 7 items for openness to experience (α=0.79), rated on a 4-point rating scale. Mean scores for personality scales were calculated for individuals with a maximum of 1 missing item in the scale, resulting in 14,549 participants with full personality data at baseline. The personality instrument was administered to half of the sample in 2006 and to the other half in 2008. Thus, the study baseline was 2006 for half of the sample and 2008 for the other half of the sample. Baseline data on other covariates were derived from the year of personality assessment.

**Height** and **weight** were self-reported by the participants, and **body mass index (BMI)** was calculated from these data with the standard formula of BMI=weight in kilograms divided by squared height in meters. **Obesity** was determined as BMI≥30. Information on **marital status** (0=single, 1=married/cohabiting) was derived from the participants’ self-reports. Data on **race/ethnicity** was based on participants’ self-reports and was coded as a dichotomous variable (0=white, non-Hispanic; 1=other). **Educational level** was determined on the basis of the highest achieved grade (0=primary education, 1=secondary education, 3=tertiary education).

Study website:

<http://hrsonline.isr.umich.edu>

Midlife in the United States (MIDUS)

The MacArthur Foundation Survey of Midlife Development in the United States (MIDUS) is based on a nationally representative random-digit-dial sample of non-institutionalized, English-speaking adults, aged 25 to 74 years, selected from working telephone banks in the coterminous United States in 1995-1996.^12,13^ The total original sample (n=7108) includes main respondents (n=3487), their siblings (n=950), a city oversample (n=757), and a twin subsample (n=1914). Data were collected in a telephone interview and with a mail questionnaire. A follow-up study of the original cohort was conducted in 2004-2005.

**Personality** was assessed at baseline with a model based on the Five Factor Model,^11^ including 5 items of extraversion (α=0.78), 4 items for neuroticism (α=0.75), 5 items for agreeableness (α=0.81), 4 items for conscientiousness (α=0.56), and 7 items for openness to experience (α=0.78). Items were rated using a 4-point rating scale on how well different adjectives described them (1=not at all, 4=a lot). Full data on personality traits at baseline were available for 6,261 participants.

**Height** and **weight** were self-reported by the participants, and **body mass index (BMI)** was calculated from these data with the standard formula of BMI=weight in kilograms divided by squared height in meters. **Obesity** was determined as BMI≥30. Information on **marital status** (0=single, 1=married/cohabiting) was derived from the participants’ self-reports. Data on **race/ethnicity** was based on participants’ self-reports and was coded as a dichotomous variable (0=white, non-Hispanic; 1=other). **Educational level** was determined on the basis of the highest achieved grade (0=primary education, 1=secondary education, 3=tertiary education).

Study website:

<http://www.midus.wisc.edu/>

National Child Development Study (NCDS)

The British National Child Development Study (also known as the 1958 British Birth Cohort Study) is a nationally representative multidisciplinary study.^14-16^ The original participants were 17,634 individuals born in England, Wales, and Scotland during one week in March 1958. Data have been collected in follow-up phases at ages 7, 11, 16, 23, 33, 42, 46, and 50. Written informed consent was obtained from the parents for childhood measurements and ethical approval for the study was obtained from the South East Multi-Centre Research Ethics Committee.

**Personality** was measured at age 50 using the 50-item Big Five model of the International Personality Item Pool (IPIP)^17^ with 10 items per personality trait rated on a 5-point rating scale. The Cronbach alpha reliabilites were 0.87 for extraversion, 0.88 for neuroticism, 0.81 for agreeableness, 0.77 for conscientiousness, and 0.78 for openness to experience. A mean score for each personality trait was calculated if no more than two items in the scale were missing, resulting in 8,697 participants with full data at baseline.

**Height** and **weight** were self-reported by the participants in the interview, and **body mass index (BMI)** was calculated from these data with the standard formula of BMI=weight in kilograms divided by squared height in meters. **Obesity** was determined as BMI≥30. Information on **marital status** (0=single, 1=married/cohabiting) was derived from the participants’ self-reports. Data on **race/ethnicity** was based on participants’ self-reports and was coded as a dichotomous variable (0=white, non-Hispanic; 1=other). **Educational level** was determined on the basis of the highest achieved grade (0=primary education, 1=secondary education, 3=tertiary education).

Study website:

<http://www.esds.ac.uk/longitudinal/access/ncds/l33004.asp>

Wisconsin Longitudinal Study (WLS)

The Wisconsin Longitudinal Study has followed a random sample of 10317 participants (5326 women, 4991 men) who were born between 1937 and 1940 and who graduated from Wisconsin high schools in 1957.^18^ After baseline data collection in 1957, survey data have been collected from the participants or their parents in 1964, 1975, 1992/3, and 2003/5. The present study used data from the 1993 follow-up. The WLS sample is broadly representative of white, non-Hispanic American men and women who have completed at least a high school education (among Americans aged 50 to 54 in 1990 and 1991, approximately 66 percent were non-Hispanic white persons who completed at least 12 years of schooling). It is estimated that about 75 percent of Wisconsin youth graduated from high school in the late 1950s – everyone in the primary WLS sample graduated from high school.

In addition to the main sample of the 1957 high school graduates, the WLS has also collected data on a selected sibling of a sample of the graduates.^19^ The data collection in adulthood has been very similar although not entirely identical for the siblings as for the graduates. For the present purposes, the sibling sample was analyzed separately from the graduate sample, because the sampling frame of the individuals for the graduate cohort and sibling cohort was considered to sufficiently to justify the decision of not combining the samples.

**Personality** data were collected in 1992-1994 via mail questionnaire including a 29-version of the Big Five Inventory (BFI).^3,4^ Participants were asked whether they agreed or disagreed that certain personality descriptions fitted themselves using a 6-point rating scale. The Cronbach alpha reliabilities were 0.76 for extraversion in graduates/0.65 in siblings for extraversion, 0.78/0.63 for neuroticism, 0.69/0.70 for agreeableness, 0.64/0.70 for conscientiousness, and 0.61/0.70 for openness to experience. A mean score for a trait was calculated if no more than 2 items of the scale were missing, resulting in 6,674 WLS graduates and 3,969 WLS siblings with full personality data at baseline.

**Height** and **weight** were self-reported by the participants, and **body mass index (BMI)** was calculated from these data with the standard formula of BMI=weight in kilograms divided by squared height in meters. **Obesity** was determined as BMI≥30. Information on **marital status** (0=single, 1=married/cohabiting) was derived from the participants’ self-reports. **Educational level** was determined on the basis of the highest achieved grade (0=primary education, 1=secondary education, 3=tertiary education).

Study website:

<http://www.ssc.wisc.edu/wlsresearch/>

References

1. Harris KM, Udry JR. National Longitudinal Study of Adolescent Health (Add Health), 1994-2008 [Computer file]. ICPSR21600-v7. Ann Arbor, MI: Inter-university Consortium for Political and Social Research [distributor], 2012-02-06. doi:10.3886/ICPSR21600.v7. 2012;

2. ESRC.Research.Centre.on.Micro-social.Change. British Household Panel Survey user manual volume A: Introduction, technical report and appendices. 2010;

3. John OP, Donahue EM, Kentle RL. The "Big Five" inventory – version 4a and 5a. 1991;

4. John OP, Naumann LP, Soto CJ. Paradigm shift to the integrative big-five trait taxonomy: History, measurement, and conceptual issues. In: John OP, Robins RW, Pervin LA, eds. Handbook of personality: Theory and research. New York, NY: Guilford Press, 2008: 114-158.

5. Wagner GG, Frick JR, Schupp J. The German Socio-Economic Panel Study (SOEP) - Scope, evolution and enhancements. Journal of Applied Social Science Studies 2007; 127:139-169.

6. DIW.Berlin. Socio-economic Pane (SOEP) (2011), Data for years 1984-2010, doi: 10.5684/sope.v27.

7. Gerlitz J-Y, Schupp J. Zur Erhebung der Big-Five-basierten Persönlichkeitsmerkmale im SOEP [The measurement of the Big Five personality traits in the SOEP]. 2005; (DIW Research Note 4/2005)

8. Wooden M, Watson N. The HILDA Survey and its contribution to economic and social research (so far). The Economic Record 83:208-231.

9. Saucier G. Mini-markers: a brief version of Goldberg's unipolar big-five markers. J Pers Assess 1994; 63:506-516.

10. Juster FT, Suzman R. An overview of the Health Retirement Study. Journal of Human Resources 1995; 30:S7-S56.

11. Lachman ME, Weaver SL. The Midlife Development Inventory (MIDI) personality scales: Scale construction and scoring. 1997;

12. Brim OG, Baltes PB, Bumpass LL et al. National Survey of Midlife Development in the United States (MIDUS), 1995-1996. [computer file]. 2007;

13. Ryff C, Almeida DM, Ayanian JS et al. Midlife Development in the United States (MIDUS2), 2004-2006 [computer file]. 2006;

14. Power C, Elliott J. Cohort profile: 1958 British birth cohort (National Child Development Study). Int J Epidemiol 2006; 35:34-41.

15. Atherton K, Fuller E, Shepherd P, Strachan DP, Power C. Loss and representativeness in a biomedical survey at age 45 years: 1958 British birth cohort. Journal of Epidemiology and Community Health 2008; 62:216-223.

16. University of London. Institute of Education. Centre for Longitudinal Studies, National Child Development Study: Childhood Data, Sweeps 0-3, 1958-1974 [computer file]. 2nd Edition. National Birthday Trust Fund, National Children's Bureau, [original data producer(s)]. Colchester, Essex: UK Data Archive [distributor], August 2008. SN: 5565.

17. Goldberg LR. A broad-bandwidth, public domain, personality inventory measuring the lower level facets of several five-factor models. ed. Personality psychology in Europe, Volume 7. Tilburg, the Netherlands: Tilburg University Press, 1999: 7-28.

18. Wisconsin Longitudinal Study Handbook (12.10.07). 2007;

19. Hauser RM, Sewell WH, Clarridge BR. The influence of family structure on socioeconomic achievement: A progress report. CDE Working Paper No 82-59 1982;

Acknowledgements

The authors wish to gratefully thank the original collectors of the data, and the ESDS (Economic and Social Data Service; <http://www.esds.ac.uk/>) and ICPSR (Inter-university Consortium for Political and Social Research; [www.icpsr.umich.edu](http://www.icpsr.umich.edu)) for making the data available.

This research uses data from Add Health, a program project designed by J. Richard Udry, Peter S. Bearman, and Kathleen Mullan Harris, and funded by a grant P01-HD31921 from the Eunice Kennedy Shriver National Institute of Child Health and Human Development, with cooperative funding from 17 other agencies. Special acknowledgment is due Ronald R. Rindfuss and Barbara Entwisle for assistance in the original design. Persons interested in obtaining data files from Add Health should contact Add Health, Carolina Population Center, 123 W. Franklin Street, Chapel Hill, NC 27516-2524 ([addhealth@unc.edu](mailto:addhealth@unc.edu)).

The data used in this study were made available through the ESRC Data Archive. The data were originally collected by the ESRC Research Centre on Micro-Social Change at the University of Essex (now incorporated within the Institute for Social and Economic Research). Neither the original collectors of the data nor the Archive bear any responsibility for the analyses or interpretations presented here.

This paper uses unit record data from the Household, Income and Labour Dynamics in Australia (HILDA) Survey. The HILDA Project was initiated and is funded by the Australian Government Department of Families, Housing, Community Services and Indigenous Affairs (FaHCSIA) and is managed by the Melbourne Institute of Applied Economic and Social Research (Melbourne Institute). The findings and views reported in this paper, however, are those of the author and should not be attributed to either FaHCSIA or the Melbourne Institute.

The research uses data from the Wisconsin Longitudinal Study (WLS) of the University of Wisconsin-Madison. Since 1991, the WLS has been supported principally by the National Institute on Aging (AG-9775 and AG-21079), with additional support from the Vilas Estate Trust, the National Science Foundation, the Spencer Foundation, and the Graduate School of the University of Wisconsin-Madison. A public use file of data from the Wisconsin Longitudinal Study is available from the Wisconsin Longitudinal Study, University of Wisconsin-Madison, 1180 Observatory Drive, Madison, Wisconsin 53706 and at http://www.ssc.wisc.edu/wlsresearch/data/. The interpretations, opinions, and inferences based on the data are solely the responsibility of the authors.

**Supplementary Figure S1.** Cross-sectional association between conscientiousness and obesity by subgroups (standardized odds ratios and 95% confidence intervals). The p-values for heterogeneity (*I*^2^) indicate statistical significance of group differences within subgroups.

**Supplementary Figure S2.** Longitudinal associations between baseline personality and subsequent obesity in the total sample (n=43,638) adjusted for baseline obesity status, the other 4 personality traits, educational level, sex, age, follow-up length, ethnicity/nationality.

**Supplementary Figure S3.** Longitudinal associations between baseline personality and subsequent obesity in non-obese individuals at baseline (n=33,981) adjusted for the other 4 personality traits, educational level, sex, age, follow-up length, ethnicity/nationality.

**Supplementary Figure S4.** Longitudinal associations between baseline personality and subsequent obesity in obese individuals at baseline (n=9,657) adjusted for the other 4 personality traits, educational level, sex, age, follow-up length, ethnicity/nationality.

**Supplementary Figure S5.** Pooled longitudinal associations between baseline personality traits and subsequent obesity when personality scores are categorized into quintiles in each study (I=Lowest quintile, the reference category, V=Highest quintile). All associations are adjusted for obesity status at baseline, sex, age, educational level, follow-up length, and ethnicity/nationality. N=43,638.

| **Supplementary Table 1.** Subgroup analyses of cross-sectional associations between personality traits and obesity. | | | | | |
| --- | --- | --- | --- | --- | --- |
|  | Extraversion | Neuroticism | Agreeableness | Conscientiousness | Openness |
| Sex |  |  |  |  |  |
| Men | 1.09 (1.04, 1.14) | 1.02 (0.97, 1.07) | 0.98 (0.91, 1.06) | 0.88 (0.83, 0.93) | 0.95 (0.91, 0.99) |
| Women | 0.99 (0.92, 1.06) | 1.03 (0.98, 1.08) | 1.06 (0.99, 1.13) | 0.81 (0.77, 0.85) | 0.95 (0.91, 1.00) |
| *I2 (p-value)* | 78% (p=0.03) | 0% (p=0.77) | 50% (p=0.16) | 79% (p=0.03) | 0% (p=0.9) |
| Age |  |  |  |  |  |
| <40 | 1.04 (1.00, 1.08) | 1.04 (0.98, 1.1) | 0.98 (0.90, 1.06) | 0.87 (0.81, 0.93) | 0.94 (0.90, 0.99) |
| 40-65 | 1.04 (0.98, 1.12) | 1.03 (0.99, 1.07) | 1.05 (0.98, 1.13) | 0.81 (0.79, 0.83) | 0.93 (0.89, 0.98) |
| 65+ | 1.03 (0.91, 1.17) | 0.96 (0.9, 1.02) | 1.03 (0.90, 1.17) | 0.84 (0.78, 0.90) | 0.97 (0.88, 1.08) |
| *I2 (p-value)* | 0% (p=0.98) | 49% (p=0.14) | 0% (p=0.41) | 56% (p=0.10) | 0% (p=0.75) |
| Education |  |  |  |  |  |
| Primary | 1.01 (0.97, 1.06) | 0.99 (0.92, 1.07) | 1.01 (0.95, 1.07) | 0.87 (0.81, 0.92) | 1.01 (0.95, 1.08) |
| Secondary | 1.03 (0.96, 1.10) | 1.01 (0.96, 1.05) | 1.00 (0.93, 1.08) | 0.84 (0.80, 0.87) | 0.98 (0.93, 1.04) |
| Tertiary | 1.04 (0.95, 1.13) | 1.01 (0.97, 1.05) | 1.07 (1.00, 1.14) | 0.81 (0.78, 0.85) | 1.03 (0.97, 1.09) |
| *I2 (p-value)* | 0% (p=0.85) | 0% (p=0.93) | 0% (p=0.39) | 33% (p=0.23) | 0% (p=0.50) |
| Geographical region |  |  |  |  |  |
| United States | 1.00 (0.91, 1.11) | 0.98 (0.95, 1.00) | 1.03 (0.91, 1.17) | 0.81 (0.77, 0.85) | 0.94 (0.87, 1.01) |
| Europe | 1.09 (1.06, 1.12) | 1.06 (1.02, 1.10) | 0.97 (0.91, 1.04) | 0.88 (0.79, 0.97) | 0.95 (0.90, 1.01) |
| Australia | 1.02 (0.97, 1.08) | 1.14 (1.07, 1.21) | 1.08 (1.01, 1.15) | 0.85 (0.8, 0.90) | 0.95 (0.90, 1.00) |
| *I2 (p-value)* | 67% (p=0.05) | 92% (p<0.001) | 60% (p=0.08) | 15% (p=0.31) | 0% (p=0.94) |
| Marital status |  |  |  |  |  |
| Married/Cohabiting | 1.05 (0.98, 1.12) | 1.03 (0.98, 1.07) | 1.03 (0.96, 1.11) | 0.82 (0.79, 0.85) | 0.95 (0.91, 0.99) |
| Single | 0.99 (0.92, 1.06) | 0.99 (0.93, 1.05) | 1.01 (0.94, 1.08) | 0.85 (0.8, 0.91) | 0.95 (0.91, 1.00) |
| *I2 (p-value)* | 36% (p=0.21) | 0% (p=0.33) | 0% (p=0.64) | 3% (p=0.31) | 0% (p=0.95) |
| Ethnicity/Nationality |  |  |  |  |  |
| Majority | 1.03 (0.97, 1.10) | 1.03 (0.98, 1.07) | 1.02 (0.95, 1.10) | 0.83 (0.79, 0.88) | 0.95 (0.91, 0.98) |
| Minority | 1.02 (0.96, 1.08) | 1.00 (0.95, 1.05) | 1.04 (0.95, 1.14) | 0.87 (0.81, 0.94) | 0.96 (0.83, 1.12) |
| *I2 (p-value)* | 0% (p=0.70) | 0% (p=0.42) | 0% (p=0.72) | 2% (p=0.31) | 0% (p=0.83) |
| Note: Values are odds ratios (and 95% confidence intervals) of logistic regression analysis predicting obesity in the different subgroups pooled across studies. Statistical significance of subgroup differences is evaluated based on heterogeneity of the effect sizes (I2) and their p-values. | | | | | |
